# The role of retrograde intraflagellar transport genes in aminoglycoside-induced hair cell death

**DOI:** 10.1242/bio.038745

**Published:** 2018-12-21

**Authors:** Tamara M. Stawicki, Tor Linbo, Liana Hernandez, Lauren Parkinson, Danielle Bellefeuille, Edwin W. Rubel, David W. Raible

**Affiliations:** 1 Program in Neuroscience, Lafayette College, Easton, PA 18042, USA; 2 Department of Biological Structure, University of Washington, Seattle, WA 98195, USA; 3 Psychology Department, Lafayette College, Easton, PA 18042, USA; 4 Virginia Merrill Bloedel Hearing Research Center, University of Washington, Seattle, WA 98195, USA

**Keywords:** Aminoglycosides, Cilia, Hair cells, Intraflagellar transport, Ototoxicity

## Abstract

Sensory hair cells are susceptible to numerous insults, including certain therapeutic medications like aminoglycoside antibiotics, and hearing and balance disorders are often a dose-limiting side effect of these medications. We show that mutations in multiple genes in both the retrograde intraflagellar transport (IFT) motor and adaptor complexes lead to resistance to aminoglycoside-induced hair cell death. These mutations also lead to defects in the entry of both aminoglycosides and the vital dye FM1-43 into hair cells, both processes that depend on hair cell mechanotransduction activity. However, the trafficking of proteins important for mechanotransduction activity is not altered by these mutations. Our data suggest that both retrograde IFT motor and adaptor complex genes are playing a role in aminoglycoside toxicity through affecting aminoglycoside uptake into hair cells.

## Abstract (precis)

**Summary:** Here we show that both retrograde intraflagellar transport motor proteins and IFT-A adaptor molecules play a role in aminoglycoside-induced hair cell death, seemingly through regulating aminoglycoside uptake.

## INTRODUCTION

During development, hair cells – the sensory cells of the auditory and vestibular systems – contain a single primary cilium on their apical surface known as the kinocilium. Primary cilia are microtubule-based structures that are believed to be important for cellular signaling in other cell types (Satir and Christensen, 2007). The kinocilium is lost in auditory hair cells of many species but always maintained in vestibular hair cells (Ernstson and Smith, 1986; Lim and Anniko, 1985; Tanaka and Smith, 1978). While mutations in cilia-associated genes have been found in multiple human patients with hearing loss (Delmaghani et al., 2016; Grati et al., 2015; Hearn et al., 2005; Ross et al., 2005), how the kinocilium affects mature hair cells has remained largely a mystery. In addition to kinocilia, hair cells also have actin-based protrusions on their apical surface known as stereocilia. Mechanotransduction, the process by which hair cells respond to stimuli, is carried out through these stereocilia in mature hair cells (Hudspeth and Jacobs, 1979). The kinocilium and cilia genes have been shown to be important for determining stereocilia polarity in mammalian auditory hair cells (Jones et al., 2008; Ross et al., 2005), however, do not appear to have this function in vestibular hair cells (Sipe and Lu, 2011) or hair cells of the zebrafish lateral line (Kindt et al., 2012; Stawicki et al., 2016). Hearing loss found in animal models with mutations in cilia-associated genes can be seen in the absence of stereocilia polarity defects (Imtiaz et al., 2018) or can develop after stereocilia defects are observed (Jagger et al., 2011) suggesting these genes have roles in hair cells independent of functions determining stereocilia polarity.

There are a number of genes required for the proper development, maintenance and function of cilia. We have previously found a role for a subset of these cilia genes in aminoglycoside-induced hair cell death (Stawicki et al., 2016). Aminoglycoside antibiotics are known to kill hair cells across a range of species and can cause hearing loss and vestibular dysfunction in human patients (Lerner et al., 1986; Moore et al., 1984). Genes important for ciliary intraflagellar transport (IFT) show a particularly large reduction in neomycin-induced hair cell death when mutated (Stawicki et al., 2016). The majority of these IFT gene mutations, including mutations of the retrograde IFT motor protein gene *dync2h1,* also lead to reductions in the amount of neomycin and FM1-43 entering hair cells. There was one exception to this: *wdr35,* a component of the IFT-A complex. Mutations of this gene showed a similar reduction in neomycin-induced hair cell death as other IFT mutants, but not as large of a change in neomycin or FM1-43 uptake (Stawicki et al., 2016).

Intraflagellar transport is the process by which proteins are trafficked along cilia and is crucial for cilia maintenance. Anterograde IFT, transport from the cell body to the ciliary tip, depends on the kinesin-2 motor and the IFT-B complex of adaptor proteins. Whereas, retrograde IFT, transport from the tip back to the base, depends on the dynein-2 motor and the IFT-A complex of adaptor proteins (Pedersen et al., 2006; Scholey, 2003). While both dynein-2 and the IFT-A complex are required for retrograde IFT it has previously been shown that mutations in genes of these two different complexes can lead to different phenotypes. For example, while *Dync2h1* mutant mice show a loss of sonic hedgehog (Shh) signaling (Huangfu and Anderson, 2005; May et al., 2005), mouse mutants in IFT-A complex genes can show excess Shh signaling (Ashe et al., 2012; Qin et al., 2011; Tran et al., 2008). Individual IFT-A gene mutants and *Dync2h1* also show different defects in cilia morphology (Cortellino et al., 2009; Liem et al., 2012; Mill et al., 2011; Ocbina et al., 2011; Tran et al., 2008) and for some cilia localized genes, transport is only affected by a subset of IFT-A gene mutations (Hirano et al., 2017; Mukhopadhyay et al., 2010). Reductions in IFT-A gene products can actually partially rescue *Dync2h1* mutant phenotypes (Liem et al., 2012; Ocbina et al., 2011).

Given these observations we wanted to further investigate whether phenotypic differences in hair cells of *dync2h1* and *wdr35* mutants were generalized to other dynein motor complex and IFT-A complex genes by looking at mutants in the dynein motor complex gene *dync2li1* and the IFT-A adaptor complex genes *ift122*, *ift140* and *wdr19*. Here we report that all these genetic mutants show comparable resistance to neomycin-induced hair cell death, and defects in neomycin and FM1-43 loading into hair cells, comparable to what was previously shown for *dync2h1* mutants. We also show that *wdr35* and *dync2h1* mutants show similar resistance to a second aminoglycoside, gentamicin. We find that that *wdr35* mutants fail to show any genetic interaction effects when combined with other IFT mutants, suggesting *wdr35* may function via a similar mechanism as other IFT genes. Lastly, we show that unlike those in anterograde IFT genes, retrograde IFT gene mutations do not lead to alterations in the localization of Usher complex genes. Overall, these results suggest that disruption of either the dynein motor or IFT-A adaptor complex will limit aminoglycoside uptake into hair cells and subsequent hair cell toxicity.

## RESULTS

### Mutations in multiple retrograde IFT genes lead to resistance to neomycin-induced hair cell death

**Fig. 1.**
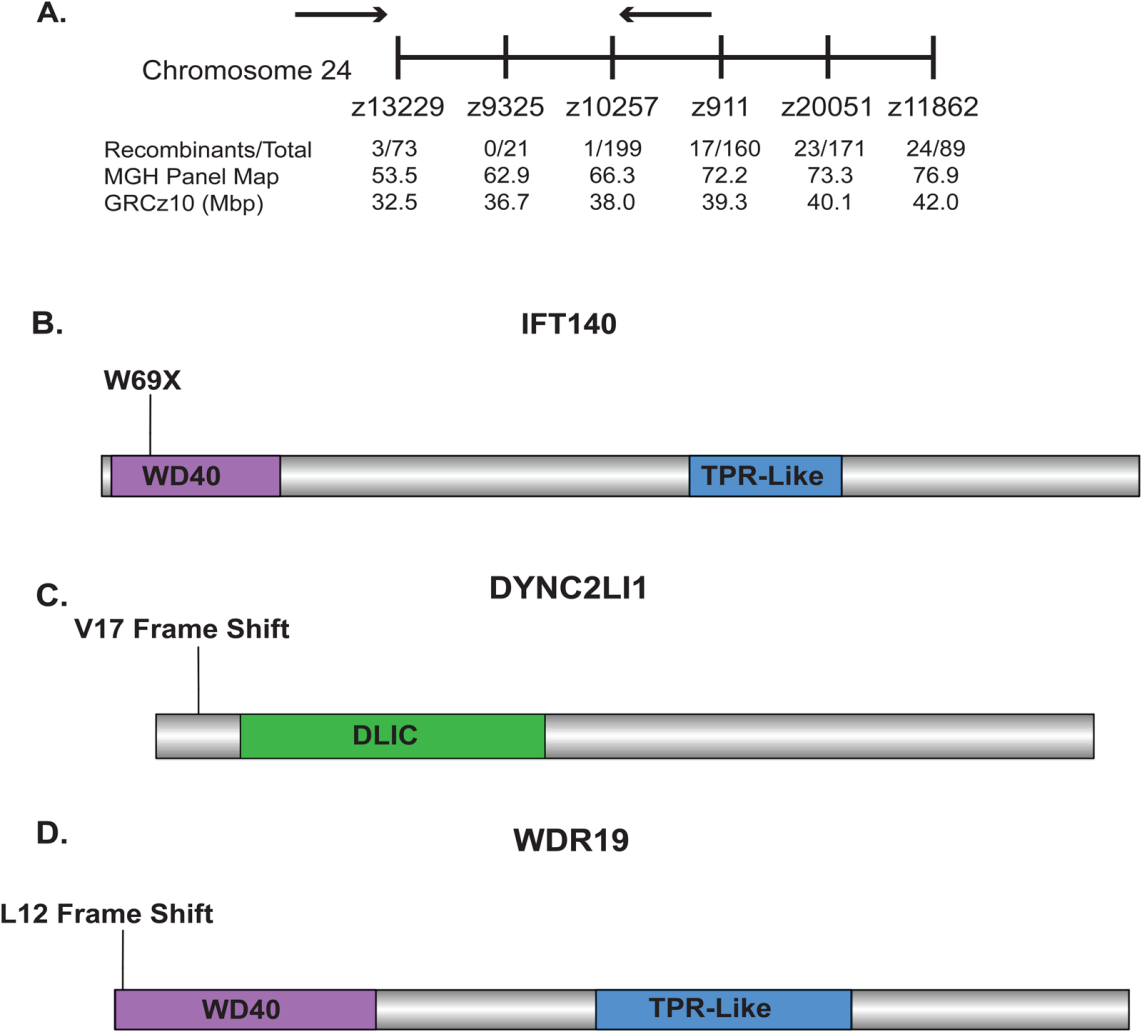
**Identification of mutations in retrograde IFT genes that confer resistance to aminoglycoside-induced hair cell death.** (A) Mutant allele *w151* mapped to a region of approximately 5.5 mega base pairs (Mbp) on chromosome 24 shown between the two arrows. The microsatellite markers used for mapping are shown, as well as the number of recombinant animals at each position. (B) Sequencing of *ift140,* a gene in the interval *w151* mapped to, found a mutation causing a premature stop codon in the WD40 repeat region in the N-terminus of the gene. TPR-like=Tetratricopeptide-like helical domain superfamily. (C) A frameshift mutation in the N-terminus of *dync2li1* after the 17th amino acid was generated using CRISPR/Cas9. DLIC is the conserved domain among dynein light intermediate chain genes. (D) A frameshift mutation in the N-terminus of *wdr19* after the 12th amino acid was generated using CRISPR/Cas9. Individual protein images are not to the same scale.

We had previously identified mutations in *dync2h1* and *wdr35*, two genes important for retrograde IFT, through a forward genetic screen for mutants resistant to neomycin-induced hair cell death (Stawicki et al., 2016). We found that another mutant identified through that screen, *w151*, mapped to a region on Chromosome 24 containing the retrograde intraflagellar transport gene *ift140* (Fig. 1A). Sequencing of *ift140* in fish with the *w151* mutant allele showed that these animals had a premature stop codon in the gene (Fig. 1B and Table S1). In addition to this mutant we also wanted to test other retrograde IFT genes. To do this we generated mutants in *dync2li1*, a dynein light intermediate chain known to associate with *dync2h1* (Hou et al., 2004; Perrone et al., 2003)*,* and the IFT-A gene *wdr19* (Fig. 1C,D and Table S1) using CRISPR mutagenesis*.* We also looked at an existing zebrafish mutant in the IFT-A gene *ift122* (Table S1, Ni et al., 2012)*.*

We found that all mutants tested had a slight but significant decrease in control hair cell numbers at 5 days post-fertilization (dpf) (Table 1). This may be due to increased cell death which has previously been shown in anterograde IFT gene mutants (Tsujikawa and Malicki, 2004). Despite this decrease in initial hair cell number all mutants also showed significantly higher hair cell numbers compared to wild-type animals following treatment with 200 µM neomycin. Whereas wild-type siblings usually had under two hair cells/neuromast remaining after neomycin treatment, mutants had on average more than five hair cells/neuromast remaining (Table 1). Both the decrease in control hair cell number and decreased amount of hair cell death seen following neomycin was comparable to what was previously shown in *dync2h1* and *wdr35* mutants (Stawicki et al., 2016).

**Table 1.**
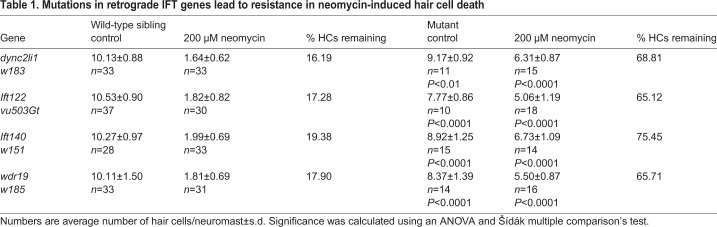
**Mutations in retrograde IFT genes lead to resistance in neomycin-induced hair cell death**

### Retrograde IFT mutants show decreased neomycin and FM1-43 loading into hair cells

**Fig. 2.**
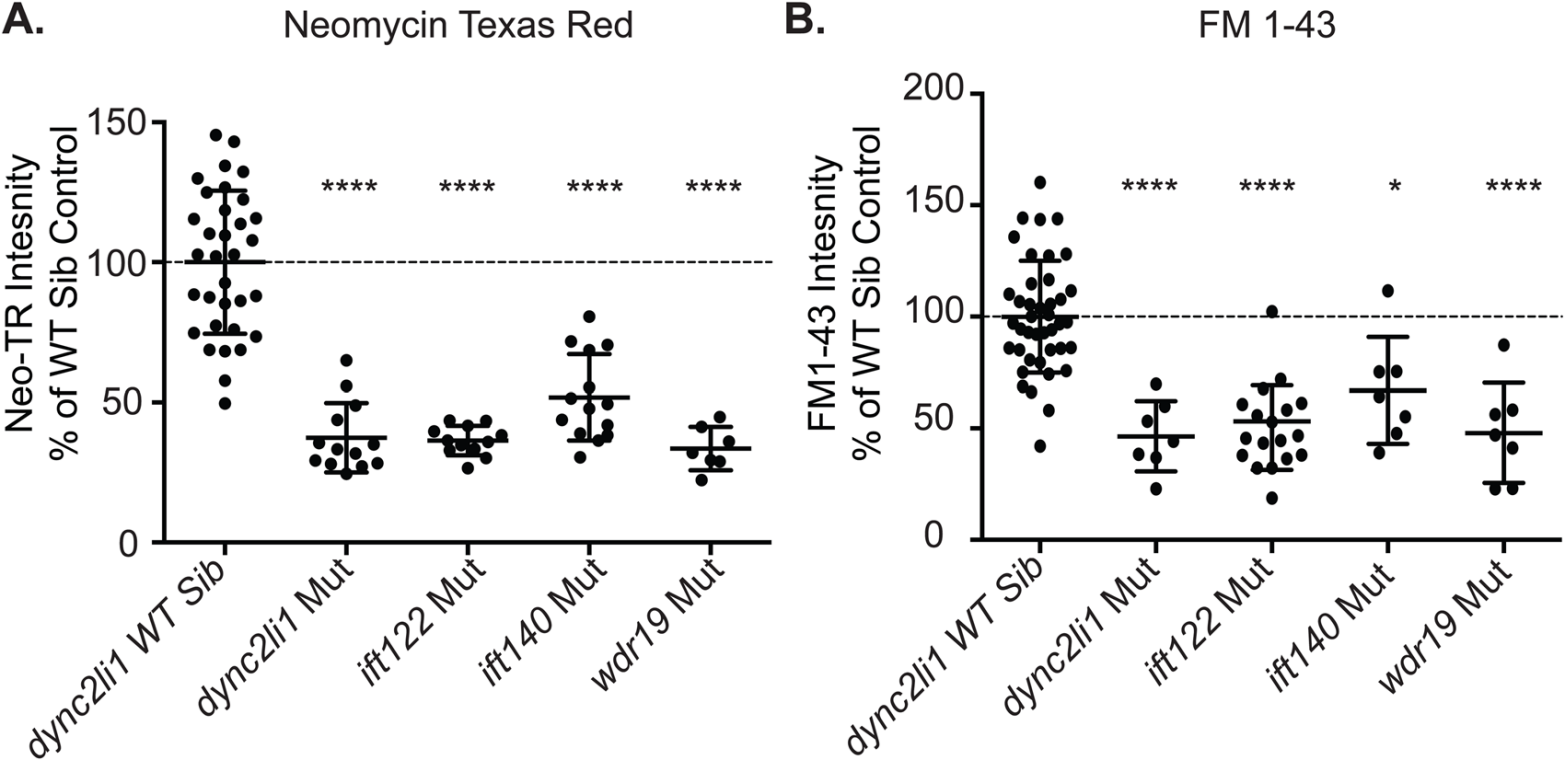
**Retrograde IFT mutants show reduced neomycin and FM1-43 uptake.** (A) Mutations in all four retrograde IFT genes show a significant decrease in neomycin-Texas Red (neo-TR) loading. ****P<0.00001. (B) Mutations in all four retrograde IFT genes show a significant decrease in rapid FM1-43 loading. *P=0.0103, ****P<0.00001. Data shows the percentage of fluorescent intensity in the cell bodies of a single neuromast as compared to the average fluorescent intensity of wild-type siblings imaged at the same time. *dync2li* wild-type sibling data is shown as representative wild-type data. Statistics are calculated using Student's or Welch's *t*-test comparing mutants to wild-type siblings. Error bars=standard deviation.

We previously showed that despite comparable levels of resistance to neomycin-induced hair cell death *dync2h1* and *wdr35* mutants showed different degrees of reduction in the loading of neomycin and FM1-43 into hair cells. *dync2h1* mutants showed a 56% reduction in neomycin-Texas Red (neomycin-TR) loading into hair cells, which was comparable to what was seen in the anterograde IFT mutants, *ift88* and *traf3ip*. In contrast *wdr35* mutants showed only a 21% reduction in neomycin loading into hair cells (Stawicki et al., 2016). This suggested that the reduction of hair cell death seen in *dync2h1* and *wdr35* may result from partially distinct mechanisms. These mutants show comparable reductions in neomycin-induced hair cell death yet *wdr35* mutants show greater neomycin-TR uptake, suggesting that this gene may play a greater role in the intracellular mechanisms of neomycin-induced hair cell death than *dync2h1.* To test whether other retrograde IFT mutants in both the dynein motor complex and IFT-A adaptor complex behaved like *dync2h1* or *wdr35* we looked at the uptake of neomycin-TR in *dync2li1*, *ift122*, *ift140* and *wdr19* mutants. We found that mutations in all these genes appeared most similar to *dync2h1* mutants with *dync2li1*, *ift122* and *wdr19* showing a greater than 60% reduction in neomycin loading into hair cells and *ift140* mutants showing a 48% reduction (Fig. 2A).

Aminoglycoside uptake into hair cells requires mechanotransduction activity (Alharazneh et al., 2011; Gale et al., 2001; Hailey et al., 2017; Marcotti et al., 2005). Mechanotransduction activity is also required for the rapid loading of the vital dye FM1-43 into hair cells (Gale et al., 2001; Meyers et al., 2003; Seiler and Nicolson, 1999). We had previously shown that differences in neomycin uptake defects between cilia mutants were paralleled by differences in FM1-43 uptake, with *dync2h1* mutants showing a significant 37% decrease in FM1-43 uptake and *wdr35* mutants not showing a significant decrease (Stawicki et al., 2016). In investigating FM1-43 uptake in our new retrograde IFT mutants we found that once again the observed defects most closely matched what was previously seen in *dync2h1* mutants. *dync2li1*, *ift122* and *wdr19* mutants showed a 50–54% decrease in rapid FM1-43 loading, whereas *ift140* mutants, similar to what was seen with neomycin-TR, showed a slightly less dramatic reduction at 33% (Fig. 2B). Combined, these results suggest that mutations in the majority of retrograde IFT gene cause significant reductions in neomycin-TR and FM1-43 uptake regardless of whether the mutated genes are part of the dynein motor complex or IFT-A complex with *wdr35* being the one anomaly.

### *dync2h1* and *wdr35* mutants show comparable resistance to gentamicin-induced hair cell death

With *wdr35* mutants showing a less dramatic reduction in neomycin uptake than other retrograde IFT mutants we wanted to further investigate whether aminoglycoside resistance seen in *wdr35* mutants was due to a distinct intracellular mechanism not shared by other IFT mutants that show more dramatic defects in aminoglycoside uptake. Mutations in *wdr35* have previously been shown to play a role in mitochondrial cell death signaling in cultured cells (Fan et al., 2012). Mitochondrial cell death pathways have also been shown to be involved in aminoglycoside-induced hair cell death (Cunningham et al., 2004; Esterberg et al., 2014; Matsui et al., 2004; Owens et al., 2007). Drugs affecting these mitochondrial cell death pathways differentially affect neomycin and gentamicin-induced hair cell death. For example, a Bax blocker has been shown to significantly protect against acute neomycin and gentamicin-induced hair cell death, while not being able to protect against continuous gentamicin-induced hair cell death. Also the p53 inhibitor pifithrin-α shows more substantial protection against continuous gentamicin exposure than acute neomycin or gentamicin exposure. (Coffin et al., 2013a,b). In contrast to this, drugs affecting hair cell mechanotransduction protect against both acute and continuous neomycin and gentamicin-induced hair cell death though higher concentrations are needed to protect against continuous gentamicin-induced hair cell death (Kirkwood et al., 2017; Owens et al., 2009). We therefore tested the response of *dync2h1* and *wdr35* mutants to both acute and chronic gentamicin treatment to see if *wdr35* showed the differential pattern of protection against these different ototoxins that has been seen with mitochondria cell death pathway blockers while *dync2h1* showed similar protection as has been seen with mechanotransduction blockers.

**Fig. 3.**
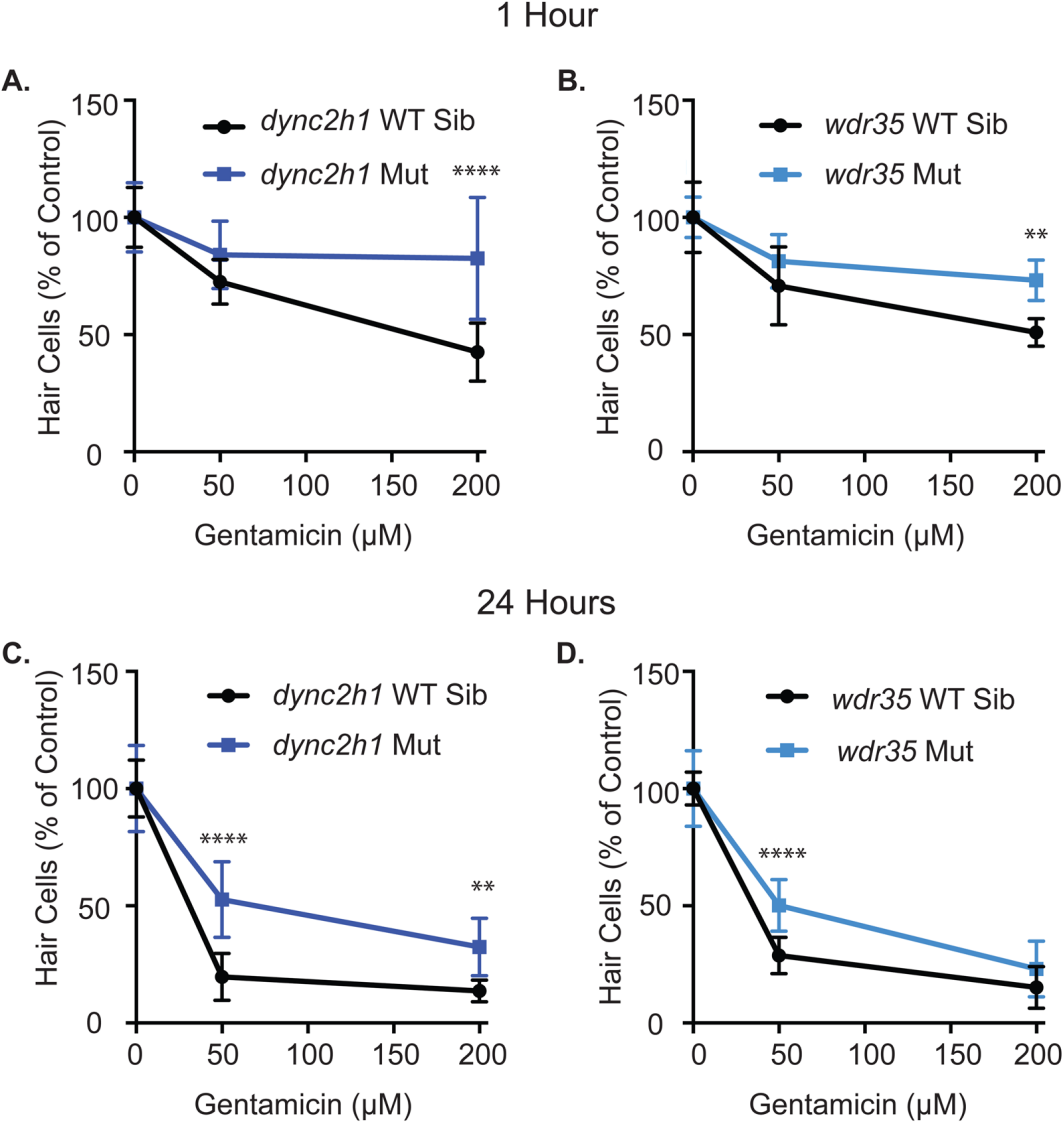
***dync2h1* and *wdr35* mutants show similar resistance to gentamicin-induced hair cell death.** Following 1 h treatment with gentamicin mutations in *dync2h1* (A) and *wdr35* (B) lead to reduced hair cell death in animals treated with 200 µM gentamicin. Following 24 h treatment with gentamicin, a mutation in *dync2h1* (C) leads to reduced hair cell death in response to both 50 and 200 µM gentamicin and a mutation in *wdr35* (D) leads to reduced hair cell death in response to 50 µM gentamicin. Data for both wild-type siblings and mutants were independently normalized to either the wild-type sibling or mutant group of control fish not treated with gentamicin. Data are displayed as mean±s.d. **P<0.01, ****P<0.00001 by two-way ANOVA and Šídák multiple comparisons test.

We found comparable changes in response to gentamicin-induced hair cell death in both *dync2h1* and *wdr35*. In response to acute 1 h gentamicin treatment no significant resistance was seen at the 50 µM dose, however, both *dynch2h1* and *wdr35* showed a significant increase in the number of hair cells remaining after treatment with 200 µM gentamicin (Fig. 3A,B). Following more chronic gentamicin treatment (24 h) resistance to 50 µM gentamicin was seen in both *dync2h1* and *wdr35* mutants. Additionally, slight resistance was seen at 200 µM in *dync2h1* mutants (Fig. 3C,D). These results suggest that as with neomycin, mutations in *dync2h1* and *wdr35* similarly affect gentamicin-induced hair cell death. While *dync2h1* mutants do appear to show slightly more resistance to gentamicin than *wdr35* mutants this could be due to the uptake differences seen in these mutants.

### *wdr35* mutants do not show genetic interactions with other IFT mutants

**Fig. 4.**
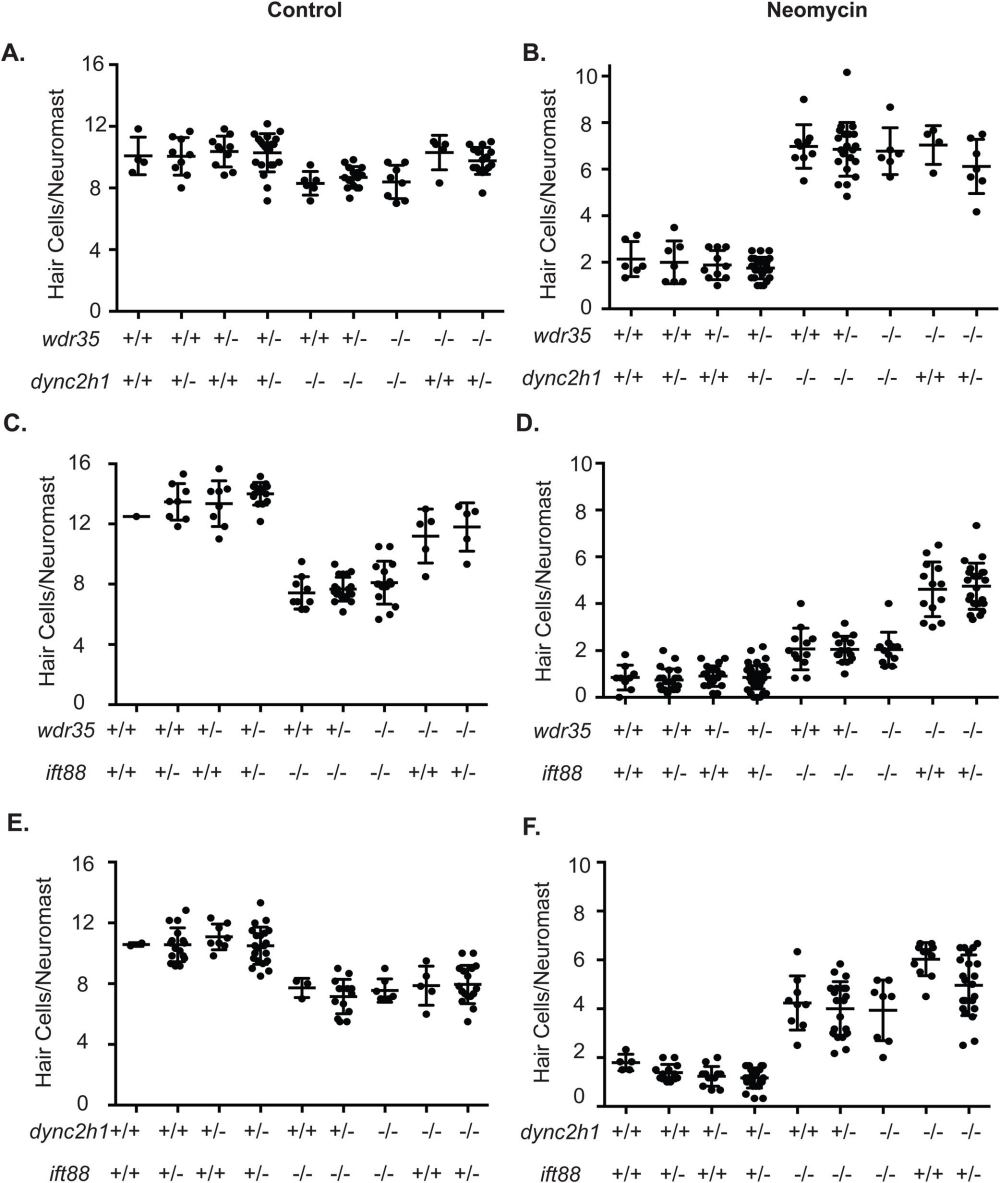
***wdr35* mutants do not show genetic interactions with other IFT gene mutants.** Hair cells per neuromast in a control situation in animals with different combinations of the *wdr35* and *dync2h1* (A), *wdr35* and *ift88* (C), and *dync2h1* and *ift88* (E) mutant alleles or following 200 µM neomycin in animals with different combinations of either *wdr35* and *dync2h1* (B), *wdr35* and *ift88* (D) and *dync2h1* and *ift88* (F) mutant alleles. There do not appear to be any genetic interactions between the different mutant alleles. +, wild-type allele; −, mutant allele. Error bars=s.d.

If *dync2h1* and *wdr35* were working through independent mechanisms in aminoglycoside-induced hair cell death one might also expect that when mutations in the two genes were combined a greater degree of resistance would be seen than in either single mutant. Synergistic phenotypes have been shown for other classes of cilia genes when mutated in combination due to presumed redundant functions (Williams et al., 2008). Alternatively, it has been shown previously that combining IFT-A mutant alleles with *Dync2h1* mutations in mice can lead to a partial rescue of the *Dync2h1* mutant phenotypes (Liem et al., 2012; Ocbina et al., 2011). To determine if there are genetic interaction effects between *dync2h1* and *wdr35* mutants we made double heterozygous animals and then looked at neomycin-induced hair cell death in the various mutant allele combinations seen in their offspring. We found combining *dync2h1* and *wdr35* mutant alleles had no effect. *dync2h1* mutants show significant reductions in control hair cell numbers and these reductions were unchanged by the addition of *wdr35* mutant alleles (Fig. 4A), and animals that were homozygous for either mutation showed a similar degree of resistance to neomycin-induced hair cell death regardless of what combination of mutant alleles for the other gene were present (Fig. 4B).

We have previously shown that a mutation in *ift88*, a gene for one of the proteins in the anterograde IFT-B complex (Pazour et al., 2000; Scholey, 2003), leads to neomycin resistance and decreases in neomycin-TR and FM1-43 uptake comparable to what is seen in *dync2h1* mutants (Stawicki et al., 2016). To see if there were genetic interaction effects between retrograde and anterograde IFT mutants we combined *ift88* mutants with both *dync2h1* and *wdr35* mutants. Again, no genetic interaction effects were observed. Decreases in control hair cell number seen in *dync2h1* and *ift88* mutants were not affected by copies of other mutant alleles (Fig. 4C,E). *ift88* mutants show slightly less neomycin-resistance than *wdr35* and *dync2h1* mutants (Stawicki et al., 2016). We found double mutants behaved like *ift88* single mutants as did single *ift88* mutants that were heterozygous for either of the other two mutations, whereas animals that were homozygous for *dync2h1* or *wdr35* showed comparable levels of neomycin resistance regardless of whether or not they had a single copy of the *ift88* mutant allele (Fig. 4D,F).

### Usher gene product localization is not affected in retrograde IFT mutants

Given the reduction in both neomycin-TR and FM1-43 loading in the majority of retrograde IFT mutants we hypothesize that mechanotransduction activity may be impaired in these mutants. However, the reason for this impairment is not clear as previous work has shown that in mature hair cells mechanotransduction is being carried out by the stereocilia, not kinocilia (Hudspeth and Jacobs, 1979). Recent work has shown that IFT genes have functions outside of cilia in multiple cell types (Cong et al., 2014; Delaval et al., 2011; Finetti et al., 2009) leaving open the possibility that these genes are functioning in hair cells independent of their role in the kinocilia. Hair cells not only contain microtubule tracks in their kinocilia, but also throughout their cytoplasm (Jaeger et al., 1994) and the anterograde IFT gene *ift88* was shown to have a cilia-independent role in the expression and trafficking of two Usher complex proteins Cadherin 23 and Harmonin (Blanco-Sánchez et al., 2014). The Usher complex is a series of proteins that interact with one another and are mutated in Usher syndrome (Adato et al., 2005). Cadherin 23 and Harmonin localize to the stereocilia and are important for hair cell mechanotransduction activity (Di Palma et al., 2001; Grillet et al., 2009; Söllner et al., 2004). Sans is another Usher complex protein that has previously been shown to associate with microtubules (Maerker et al., 2008) and thus also could have the potential to be influenced by IFT genes.

**Fig. 5.**
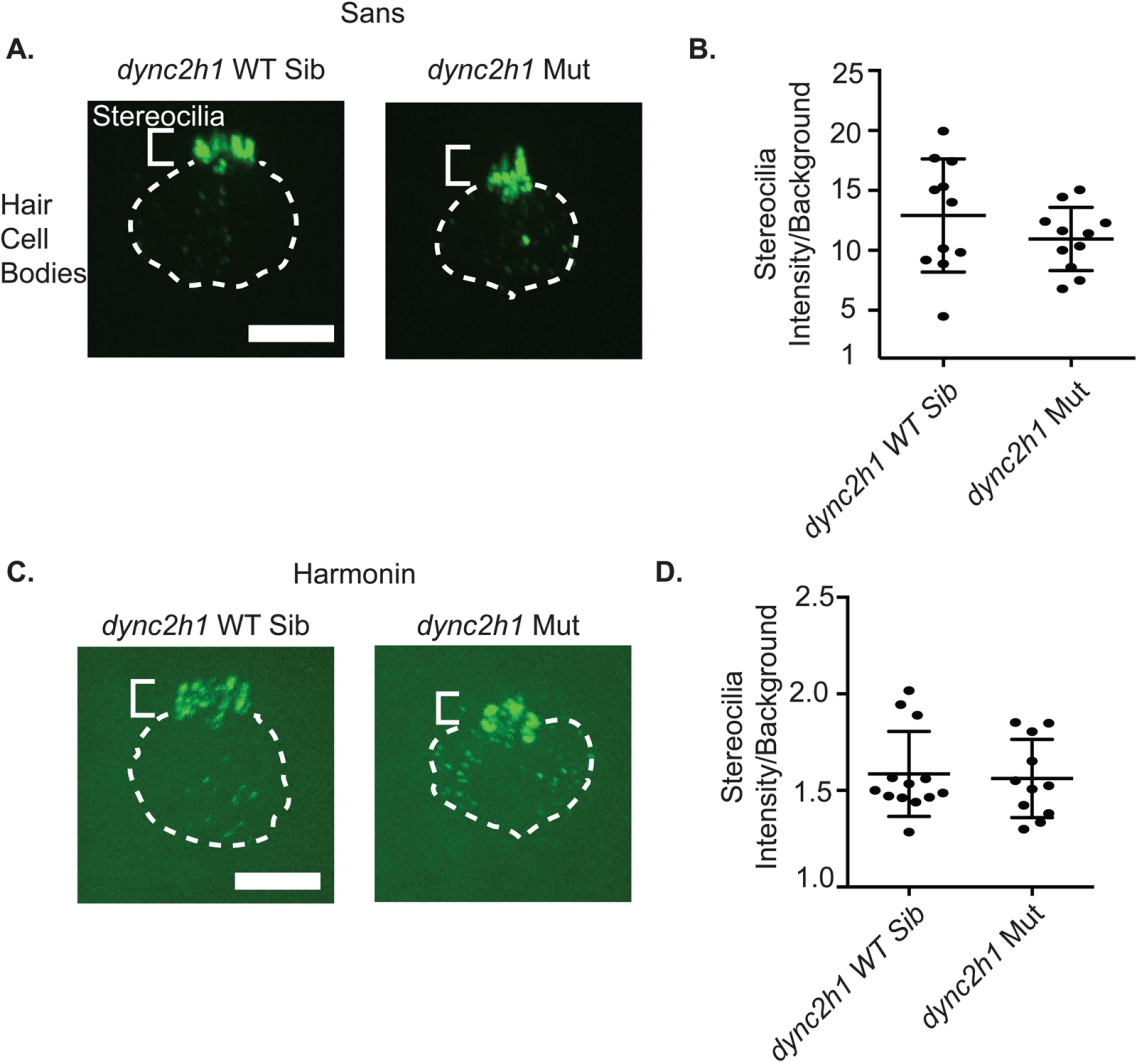
**Retrograde IFT mutants do not show mislocalization of usher gene products.** (A) Images of neuromasts expressing *pmyo-6:eos:sans* in *dycn2h1* wild-type siblings and *dync2h1* mutants. Sans localizes primarily to the stereocilia in both genotypes. The smaller upper brackets show the area of the figure where the stereocilia are located whereas the dashed lines show where the hair cell bodies are located. Scale bar: 10 µM. (B) Quantification of *pmyo-6:eos:sans* fluorescence intensity in the stereocilia normalized to background fluorescence. (C) Images of neuromasts expressing *pmyo-6:harmonin:eos* in *dync2h1* wild-type siblings and *dync2h1* mutants. Harmonin localizes primarily to the stereocilia in both genotypes. (D) Quantification of *pmyo-6:harmonin:eos* fluorescence intensity in the stereocilia normalized to background fluorescence. Error bars=s.d.

To test if retrograde IFT genes were similarly important for trafficking and localization of usher gene complex proteins we expressed constructs containing Harmonin and Sans conjugated with the fluorescent protein EOS in hair cells. We found that both Sans and Harmonin Eos fusion proteins localized primarily to the stereocilia in wild-type animals and that this localization was unaltered in *dync2h1* mutants (Fig. 5A,C). Nor was there a significant decrease in expression of either protein in *dync2h1* mutants (Fig. 5B,D).

## DISCUSSION

It has previously been shown that the dynein retrograde IFT motor complex can have distinct functions in cells from the IFT-A retrograde IFT adaptor complex potentially due to a role for IFT-A complex genes in anterograde IFT (Liem et al., 2012; Ocbina et al., 2011) or differences in cargo associated with specific IFT-A molecules (Mukhopadhyay et al., 2010). Our previous results suggested that this may be the case for the role of these retrograde IFT gene types in aminoglycoside-induced hair cell death where mutations in *dync2h1* but not *wdr35* showed more dramatic reductions in the uptake of aminoglycosides and the vital dye FM1-43 into hair cells (Stawicki et al., 2016). Here, we find that this hypothesis did not hold up upon examining mutations in additional retrograde IFT genes. We found that mutations in the retrograde IFT motor complex gene *dync2li1*, and mutations in the IFT-A adaptor complex genes *ift122*, *ift140* and *wdr19* all lead to comparable levels of resistance to aminoglycoside-induced hair cell death and a reduction in both aminoglycoside and FM1-43 uptake into hair cells comparable to what is seen with mutations in the motor gene *dync2h1*.

This led to the question of whether *wdr35* alone was acting more distinctly than the other retrograde IFT genes. While we cannot definitively rule out that *wdr35* is acting through a distinct mechanism, as we do not know the exact mechanism of its action, we did not find any additional evidence to support that it does. There were no genetic interaction effects between *wdr35* and *dync2h1.* Nor did we find any genetic interaction effects between either *wdr35* or *dync2h1* and the anterograde IFT-B complex gene *ift88.* This is different from what has been seen in other systems where IFT genes are thought to have different functions (Liem et al., 2012; Ocbina et al., 2011). Additionally, both *dync2h1* and *wdr35* mutants showed similar resistance to both short and long-term gentamicin-induced hair cell death despite the fact that these processes are known to involve slightly different intracellular cell death pathways (Coffin et al., 2013a,b).

Both *dync2h1* and *wdr35* mutants showed less resistance against gentamicin-induced hair cell death than had previously been seen with neomycin (Stawicki et al., 2016). Previous work in the mammalian system has shown that even in cases of prolonged exposure to gentamicin its entry is dependent on mechanotransduction activity rather than endocytosis (Alharazneh et al., 2011), so we do not believe this is due to gentamicin entering the cells in a mechanotransduction-independent manner in the case of continuous treatment. However, none of the tested retrograde IFT mutants completely block mechanotransduction-dependent processes like rapid FM1-43 or neomycin-TR entry (Stawicki et al., 2016), thus over a prolonged treatment period sufficient levels of aminoglycosides may be able to enter hair cells to cause significant hair cell death even if mechanotransduction activity is impaired. This is in agreement with previous work that has shown higher concentrations of mechanotransduction blocking drugs are needed to protect against continuous gentamicin-induced hair cell death as compared to acute neomycin-induced hair cell death (Kirkwood et al., 2017; Owens et al., 2009).

This leaves open the question of why uptake defects in *wdr35* are not as dramatic as those seen in other IFT gene mutants. Biochemical evidence suggests that IFT122, IFT140 and IFT144 (WDR19) make up a core component of the IFT-A complex whereas IFT121 (WDR35) is a peripheral component (Behal et al., 2012; Hirano et al., 2017). It is still unclear what this means functionally. Disruption of the core components appears to have a more dramatic effect on trafficking of certain proteins than peripheral components (Hirano et al., 2017; Mukhopadhyay et al., 2010), however, other studies have shown more dramatic or additional phenotypes in peripheral IFT-A component mutants (Fu et al., 2016; Liem et al., 2012; Mill et al., 2011). As we see more dramatic effects on uptake following mutations of the core complex genes *ift122*, *ift140* and *wdr19* than the peripheral gene *wdr35* our results would be consistent with the former work. It is conceivable that mislocalization of a molecule dependent on IFT trafficking is responsible for the defects in aminoglycoside uptake seen in IFT mutants and the localization of this molecule is more severely disrupted following mutations of core IFT-A complex or motor protein genes than it is by mutations in peripheral components.

Another open question is why aminoglycoside and FM1-43 uptake are reduced in IFT mutants. Previous work showed that the anterograde IFT protein IFT88 was important for the trafficking of Cadherin 23 and Harmonin (Blanco-Sánchez et al., 2014), two usher complex proteins important for hair cell mechanotransduction activity (Di Palma et al., 2001; Grillet et al., 2009; Söllner et al., 2004). However, we did not see mislocalization of harmonin or sans, another usher complex gene, in animals with a mutation in the retrograde IFT gene *dync2h1.* Another possibility is that uptake is reduced due to the absence of the kinocilia seen in IFT mutants (Stawicki et al., 2016). Aminoglycosides can be seen entering the kinocilia of hair cells at the same time that they are entering the stereocilia and before they enter the cell body suggesting the kinocilia as a possible route of entry (Hailey et al., 2017). Also while kinocilia have been shown to not play a role in mechanotransduction in mature hair cells (Hudspeth and Jacobs, 1979) they do appear to play a role in reverse polarity lateral line hair cell mechanotransduction early in development (Kindt et al., 2012). It is possible that disruption of this early mechanotransduction activity may disrupt the development of mature mechanotransduction in these mutants. It has been shown that mutations in *Piezo2,* which appears to be responsible for the reverse polarity currents seen in mouse hair cells, lead to hearing defects in mature animals (Wu et al., 2017). However, if the uptake defects we observe were due to simply the loss of the kinocilia, this does not explain why the defects are less severe in *wdr35* mutants where the kinocilia is also absent (Stawicki et al., 2016). Another possibility is that IFT genes are influencing aminoglycoside uptake through regulating a cell signaling process or cellular cytoskeleton dynamics. Multiple human deafness genes are cilia localized genes implicated in these processes (Delmaghani et al., 2016; Grati et al., 2015; Imtiaz et al., 2018). IFT genes have also been shown to influence these processes in other cell types independent of cilia (Cong et al., 2014; Delaval et al., 2011; Finetti et al., 2009). As a number of IFT genes have been shown to be expressed in mature mammalian auditory hair cells after the loss of the kinocilia (Liu et al., 2014) this opens the possibility that the role of IFT genes in aminoglycoside uptake is not restricted to hair cells with kinocilia.

Our findings show a broad role for retrograde IFT genes in aminoglycoside uptake and toxicity. Further studies will investigate the role these genes play in hair cell signaling processes and cytoskeletal dynamics to try to uncover the mechanism by which they are acting.

## MATERIALS AND METHODS

### Animals

All experiments used 5 days post-fertilization (dpf) *Danio rerio* (zebrafish) larvae. Mutations used in these studies are summarized in Table S1. Mutant alleles were maintained as heterozygotes in the *AB strain background and all experiments were carried out in this strain background. Wild-type siblings refer to fish that were both homozygous wild type and heterozygous for the mutant allele as no significant difference was observed between these two groups. Larvae were raised in embryo media (EM) consisting of 1 mM MgSO_4_, 150 μM KH_2_PO_4_, 42 μM Na_2_HPO_4_, 1 mM CaCl_2_, 500 μM KCl, 15 mM NaCl and 714 μM NaHCO_3._ The University of Washington or Lafayette College Institution Animal Care and Use Committee approved all experiments. Mutant and transgenic strains used in this study are available upon request.

### CRISPR mutagenesis

To generate CRISPR mutants guide RNAs (gRNAs) for two different target sites were generated for each gene. For *dync2li* those target sites were GGAGAGCAGGACTGATGAAG and GAAGAAGACTGTTCTCTGCG. For *wdr19* those target sites were GGTCTTCGCTGCCCAACGCC and GGAGATGGCTATATCATGAT. Targets were selected using the design tool at http://crispr.mit.edu. Cas9 mRNA and gRNA were synthesized as previously described (Shah et al., 2015). Embryos were injected with approximately 1nl of a solution containing 200 ng/µl of Cas9 mRNA and 50 ng/µl each of the two gRNAs. Transmission of a genetic change at the gRNA target site was screened for by performing a PCR using primers flanking the target and running the product on a 3% lithium borate gel (Brody et al., 2004) to look for size changes. Experiments were performed on larvae in the F3 generation.

### Genetic screening

F2 mutant families were generated and screened as previously described (Owens et al., 2008; Stawicki et al., 2016).

### Genetic mapping

To determine the DNA mutation in the *w151* allele originally identified through phenotyping screening, genetic mapping was carried out using the Wik strain. *AB/Wik hybrid carriers of the mutant allele were incrossed to generate progeny for linkage mapping analysis. Mutant and wild-type fish were selected based on the amount of hair cell death seen in response to 200 µM neomycin. Microsatellite markers for each chromosome (Knapik et al., 1998; Shimoda et al., 1999) were amplified by PCR and tested for cosegregation with mutant phenotypes. Pools of 20 wild-type siblings and mutants were initially used for bulk segregant analysis. Markers cosegregating with the mutant allele were then further evaluated with individual DNA from 204 mutants and 69 wild-type siblings. To sequence candidate genes following linkage mapping RNA was isolated from pools of 20 wild-type siblings or mutant embryos using TRIzol Reagent (Ambion), and cDNA was prepared using SuperScript III Reverse Transcriptase (Invitrogen). Genes were amplified by PCR from the resultant cDNA and then sent to Eurofins MWG Operon for sequencing.

### Aminoglycoside treatment

For neomycin experiments fish were treated with 200 µM of neomycin (Sigma-Aldrich) dissolved in EM for 30 min at 28.5°C. They were then washed three times in EM and left to recover for 1 h. Animals used for genetic mapping were screened for neomycin resistance using the vital dye DASPEI {2-[4-(dimethylamino)styryl]-N-ethylpyridinium iodide} (Molecular Probes). They were exposed to DASPEI at a final concentration of 0.05% for 15 min and then washed twice in EM before analyzing. Neuromasts SO1, SO2, IO1, IO2, IO3, IO4, O2, M2, MI1 and MI2 were scored as previously described (Harris et al., 2003). Animals with a score of eight or higher were considered resistant and collected as mutants whereas animals with a score of two or lower were collected as wild-type siblings. Animals used for hair cell counts were fixed for immunostaining. For each animal, hair cells were counted in the OP1, M2, IO4, O2, MI2 and MI1 neuromasts (Raible and Kruse, 2000) and then an average number of hair cells/neuromasts was calculated.

For gentamicin experiments fish were treated with either 50 or 200 µM of gentamicin (Sigma-Aldrich) dissolved in EM for either 1 or 24 h at 28.5°C. They were then washed three times in EM and immediately fixed for immunostaining. Hair cells were counted in the OP1, M2, IO4, O2, MI2 and MI1 neuromasts (Raible and Kruse, 2000) and then an average number of hair cells/neuromast was calculated. Hair cell numbers following gentamicin treatment were normalized to the average hair cells/neuromast of untreated control fish of the same genotype.

For *dync2h1*, wild-type siblings and mutants were separated before the experiments based on the mutant's secondary body morphology phenotype and 10 animals from each group were used for each condition. For all other mutants 48 animals/condition were used for single mutant experiments and 96 animals/condition for double mutant experiments. Animals were genotyped after counting hair cells to separate wild-type siblings from mutants. In some cases, animals were lost during the staining and genotyping procedures and therefore the final sample size was reduced.

### Immunohistochemistry

Fish used for immunohistochemistry were fixed for either 2 h at room temperature or overnight at 4°C in 4% paraformaldehyde. Antibody labeling was carried out as previously described (Stawicki et al., 2014). Fish used for hair cell counts were labeled with either a mouse anti-parvalbumin primary antibody (Millipore, MAB1572) diluted at 1:500 in antibody block (5% heat-inactivated goat serum in PBS, 0.2% Triton, 1% DMSO and 0.2% BSA) or a rabbit anti-parvalbumin primary antibody (Thermo-Fisher Scientific, PA1-933) diluted at 1:1000 in antibody block.

### Uptake experiments

Fish were treated with either 2.25 µM FM1-43 FX (Molecular Probes) for 1 min or 25 µM neomycin-Texas Red (TR) for 5 min. Neomycin-TR was made as previously described (Stawicki et al., 2014). Fish were then washed three times in EM and anesthetized with MS222 for imaging. Images were obtained and analyzed using SlideBook software on a Marianas spinning disk confocal system (Intelligent Imaging Innovations). For each animal a single neuromast was imaged and analyzed. A stack of 30 1 µm optical sections was obtained for that neuromast and maximum projection images were analyzed. The average fluorescent intensity of the cell bodies of the entire neuromast was calculated and divided by the background fluorescence of the image. The fluorescence measurements of mutant neuromasts were then normalized to the average fluorescent intensity of wild-type sibling controls imaged on the same day with the same imaging parameters. Forty-eight animals were imaged for each condition and then genotyped after imaging to determine which were wild-type siblings and which mutants.

### Usher gene localization

*sans* and *harmonin* were cloned into Gateway entry vectors (Thermo-Fisher Scientific) using *AB zebrafish cDNA. The *pmyo-6b:eos:sans* and *pmyo-6b:harmonin:eos* constructs were then constructed in a Tol2 transposon backbone (Kwan et al., 2007) using standard gateway cloning techniques. DNA constructs were then injected into single cell embryos at 200 pg along with 40 ng of transposase mRNA and a co-injection marker expressing GFP in the heart was used to identify transgene carriers. These fish were grown to adulthood and screened for germline incorporation of the transgene generating the stable lines *Tg(pmyo-6b:eos:sans)^w219^* and *Tg(pmyo-6b:harmonin:eos)^w220^* in the heterozygous *dync2h1* mutant background. For experiments these transgenic animals were crossed to *dync2h1* heterozygotes without the transgene and wild-type siblings and mutants were separated based on their body morphology. Images were obtained and analyzed using SlideBook software on a Marianas spinning disk confocal system (Intelligent Imaging Innovations). A stack of 30 1 µm optical sections was obtained for each neuromast and maximum projection images were analyzed. The average fluorescent intensity of the stereocilia was calculated and divided by the background fluorescence of the image. One neuromast was imaged for each animal.

### Statistical analysis

All statistics were calculated using GraphPad Prism software (version 6.0). Mean and standard deviation is shown for all data. In most cases individual data points are shown as well.

## Supplementary Material

Supplementary information
