# Supplementary material for: The role of retrograde intraflagellar transport genes in aminoglycoside-induced hair cell death
Source: Biol Open. 2018 Dec 21;8(1):bio038745. doi: 10.1242/bio.038745 (PMC6361216; doi:10.1242/bio.038745)
Supplement: Supplementary information [file biolopen-8-038745-s1.pdf]

Table S1. Mutant Strains used in this study

Gene	Allele	Nucleotide Change	Protein Change	How Generated	Reference
dyn2li1	*w183*	26 bp deletion after g53	Frame shift after V17	CRISPR	
dync2h1	*w46*	g1452a	W484Stop	ENU	(1)
ift88	*tz288*	t779a	L260Stop	ENU	(2,3)
ift122	*vu503Gt*	Exon 10		Transgenic Insertion	(4)
ift140	*w151*	g207a	W69Stop	ENU	
wdr19	*w185*	5 bp deletion after t458	Frame shift after L12	CRISPR	
wdr35	*w150*	t2426a	L809Stop	ENU	(1)

1. Stawicki TM, Hernandez L, Esterberg R, Linbo T, Owens KN, Shah AN, et al. Cilia-associated genes play differing roles in aminoglycoside-induced hair cell death in zebrafish. G3 Genes, Genomes, Genet. 2016;6(7):2225–35.
2. Brand M, Heisenberg CP, Warga RM, Pelegri F, Karlstrom RO, Beuchle D, et al. Mutations affecting development of the midline and general body shape during zebrafish embryogenesis. Development. 1996 Dec 1;123(1):129–42.
3. Tsujikawa M, Malicki J. Intraflagellar Transport Genes Are Essential for Differentiation and Survival of Vertebrate Sensory Neurons. Neuron. 2004 Jun 10;42(5):703–16.
4. Ni TT, Lu J, Zhu M, Maddison LA, Boyd KL, Huskey L, et al. Conditional control of gene function by an invertible gene trap in zebrafish. Proc Natl Acad Sci U S A. 2012 Sep 18;109(38):15389–94.
